# Immune Checkpoints Are New Therapeutic Targets in Regulating Cardio-, and Cerebro-Vascular Diseases and CD4^+^Foxp3^+^ Regulatory T Cell Immunosuppression

**DOI:** 10.53941/ijddp.2024.100022

**Published:** 2024-11-26

**Authors:** Ying Shao, William Y. Yang, Gayani Nanayakkara, Fatma Saaoud, Mohammed Ben Issa, Keman Xu, Yifan Lu, Xiaohua Jiang, Sadia Mohsin, Hong Wang, Xiaofeng Yang

**Affiliations:** 1 Lemole Center for Integrated Lymphatics and Vascular Research, Department of Cardiovascular Sciences, Lewis Katz School of Medicine at Temple University, Philadelphia, PA19140, USA; 2 Center for Metabolic Disease Research, Department of Cardiovascular Sciences, Lewis Katz School of Medicine at Temple University, Philadelphia, PA19140, USA; 3 Eccles Institute of Human Genetics, University of Utah, Salt Lake City, UT84112, USA; 4 Aging + Cardiovascular Discovery Center (ACDC), Department of Cardiovascular Sciences, Lewis Katz School of Medicine at Temple University, Philadelphia, PA19140, USA

**Keywords:** immune checkpoints (ICPs), CD4^+^Foxp3^+^ regulatory T cells (Tregs), T cells, innate immune cells, cardiovascular diseases, cerebrovascular diseases

## Abstract

Although previous reviews explored the roles of selected immune checkpoints (ICPs) in cardiovascular diseases (CVD) and cerebrovascular diseases from various perspectives, many related aspects have yet to be thoroughly reviewed and analyzed. Our comprehensive review addresses this gap by discussing the cellular functions of ICPs, focusing on the tissue-specific and microenvironment-localized transcriptomic and posttranslational regulation of ICP expressions, as well as their functional interactions with metabolic reprogramming. We also analyze how 14 pairs of ICPs, including CTLA-4/CD86-CD80, PD1-PDL-1, and TIGIT-CD155, regulate CVD pathogenesis. Additionally, the review covers the roles of ICPs in modulating CD4^+^Foxp3^+^ regulatory T cells (Tregs), T cells, and innate immune cells in various CVDs and cerebrovascular diseases. Furthermore, we outline seven immunological principles to guide the development of new ICP-based therapies for CVDs. This timely and thorough analysis of recent advancements and challenges provide new insights into the role of ICPs in CVDs, cerebrovascular diseases and Tregs, and will support the development of novel therapeutics strategies for these diseases.

## 1. Introduction

Immune checkpoints (ICPs) are a group of immune regulatory proteins located on the plasma membranes of antigen-presenting cells (APCs, which act as ligands) and T cells (which act as receptors). These proteins are essential for maintaining self-tolerance (i.e., preventing autoimmunity) and for controlling the intensity and effectiveness of T cell immune responses, thereby protecting tissues from damage [1]. T cell responses are initiated by ligand-receptor interactions between T cell and APCs, where T cell receptors (TCR) on T cells recognize the complexes of major histocompatibility (MHC)-antigen epitopes presented by APCs, serving as the initial T cell activation signal 1 [2–12]. Classic ICPs include co-inhibitory receptors (CIRs), which suppress T-cell functions, and co-stimulatory receptors (CSRs), which promote T cell activation. The amplitude, quality, and duration of T cell responses are governed by a balance between signals from co-stimulatory and co-inhibitory receptors (ICPs), which serve as the T cell activation signal 2.

Previously, we reported several noteworthy findings regarding the tissue expressions and regulatory mechanisms of ICPs, including: (1) ICPs (co-signaling receptors), which include CSRs and CIRs, exhibit differential expression across 32 human tissues; (2) two CSRs, CD40 and CD70, play crucial roles in regulating all four key functions of T cells, including priming, differentiation of T helper cell, effector function, and memory; (3) human tissues display distinct patterns in regulating co-signaling pathways involved in these T cell functions; (4) the proinflammatory caspase-1 activating protein complex, known as the inflammasome [13–15], regulates reverse signaling via co-signaling receptors (both CSRs and CIRs); (5) vascular endothelial cell (EC) growth factor receptor 3 (VEGFR3) influences the expression of co-signaling receptor, suggesting a role for ICPs in lymphangiogenesis [16,17]; (6) the ratio of S-adenosylhomocysteine (SAH) [18] to S-adenosylmethionine (SAM)-determines methylation status [19–21], which in turn regulates the expression of co-signaling receptors in mice; (7) high expression of co-signaling receptors is correlated with specific immune cell markers, including the macrophage marker F4/80, B cell marker CD20, and T cell antigen receptor components CD3g and CD3e, except CD11C; (8) reverse signaling through many CSRs plays an important role in enhancing the polarization of pro-inflammatory type 1 macrophages (M1); (9) both CSRs and CIRs are involved in regulating immune tolerance, anti-inflammatory responses, and the resolution of inflammation; (10) CSRs and CIRs also regulate the expression of EC adhesion molecules and vascular smooth muscle cells (VSMC) phenotypic markers, contributing to innate immunity functions; and (11) CSRs and CIRs possess intracellular domains with phosphorylation sites capable of reverse signaling [22].

Additionally, we observed that CD274 is highly expressed in the intestine, lung, liver, and spleen compared to adipose tissue. This higher expression correlates with elevated levels of markers for three subgroups of proinflammatory M1-like macrophages (M1-, M4-, and Mox-macrophages) in these tissues, suggesting that CD274^+^ cells may play a role in suppressing proinflammatory M1-like macrophages in tissues with high inflammatory potential [15,23]. The association between ICPs and the development of cardiovascular diseases (CVDs), stroke, and cerebrovascular diseases become a notable concern [24–31]. This suggests that ICPs have a profound impact on the overall health of the cardiovascular and cerebrovascular system. This influence is further underscored by the roles of immune cells, including T cells and APCs, which are influenced by inflammatory signals and contribute to the initiation and progression of CVDs [32–34]. Additionally, studies using gain-of-function and loss-of-function mouse models, as well as specific human patient populations, have demonstrated that ICPs and their ligands, including PD-1/PD-L1, CTLA-4/CD80 and CD86, ICOSL (CD275)/ICOS (CD278), CD40/CD40L (CD154), OX40 (CD134)/OX40L (CD252), GITR (TNFRSF18)/GITRL (TNFSF18)/ and TIM (HAVCR2, CD366) axes, are associated with an increased risk of vein graft disease [35], graft arterial disease [36], atherosclerosis [37–40], coronary heart disease, ischemic stroke [25,26], myocarditis [41], cardiomyopathy [42], and heart failure [43]. Notably, immunomodulatory therapy using canakinumab (a monoclonal antibody targeting interleukin-1β (IL-1β)) has been successfully implemented in the treatment of atherosclerosis [44], highlighting the significance of targeting immune-enhancing molecules.

Both co-inhibitory and co-stimulatory receptors, along with their ligands, exhibit considerable diversity in expression, structure, and function, with their roles being predominantly context-dependent [45]. For example, CD80/CD86 co-stimulation plays a role in promoting graft arterial disease after heart transplantation. Blocking CD80/CD86 with the agonist CTLA-4 immunoglobulin following arterial graft transplantation improves long-term graft survival and reduces the development of graft arteriosclerosis by decreasing T cells and macrophages activation [46]. However, the differential expression patterns and dynamics of CD80 and CD86 (ligands of CTLA-4) during alloimmune responses—where CD86 is upregulated in the early stages and CD80 is upregulated later [36,47]—results in distinct outcomes between CD80/CD86 blockade and selective CD80 blockade [48]. Blocking CD80/CD86 at a later stage or selectively blocking CD80 alone can reduce the progression of graft arterial disease, but early graft loss was not prevented by CD80 blockade alone, unlike the more effective CD80/CD86 double blockade. These findings underscore the importance of understanding the diverse roles of ICP axes across different disease stages and cell types in the context of CVDs and cerebrovascular diseases.

Many of our previous studies, along with those of others, have provided evidence supporting the involvement of ICP axes in the development of CVDs. Some of these key findings include: (1) T cells mediate the pathogenesis of CVDs through forward signaling [22], where APCs regulate both effector T cells (Teffs) and CD4^+^Foxp3^+^ regulatory T cells (Tregs) [45,49–52]; (2) APCs, including monocytes, macrophages [23,53,54], natural killer (NK) cells, dendritic cells (DCs), and other unconventional APCs such as endothelial cells (ECs) [55–58], VSMCs [59,60], and cardiomyocytes, present antigens to T cells [61–64]. Specifically, Tregs regulate the functions of these APCs through a process known as reverse signaling; and (3) The modulation of inflammatory signaling plays a critical role in the pathogenesis of CVDs and cerebrovascular diseases, potentially involving both dependent and independent receptor-ligand interactions of ICPs [65]. Notably, several excellent reviews have explored these critical topics from various perspectives [66,67]. However, many related issues regarding the involvement of ICPs in the progression of CVDs and cerebrovascular diseases have yet to be thoroughly reviewed and analyzed. This comprehensive review will address these gaps.

An increasing number of receptors and their corresponding ligands are continually being discovered and added to the immune checkpoint family (Table 1) [22,68]. Ongoing research aims to understand the binding affinities and cell type localization of these newly discovered ICP receptor-ligand pairs. To effectively utilize ICP molecules as therapeutic targets in the context of CVDs and cerebrovascular diseases, it is crucial to deepen our understanding of the activating and inhibitory signals regulated by these new ICP receptor-ligand axes in various cellular systems.

## 2. Immune Checkpoints Play Both Physiological and Pathological Roles and Are Closely Linked to Metabolic Reprogramming

ICPs play crucial roles in facilitating self-renewal [69] and transdifferentiation including epithelial-mesenchymal transition (EMT) [70], which is similar to the phenotype switch [71] from VSMCs to mesenchymal-like VSMCs and the transdifferentiation [72] of VSMCs to endothelial-mesenchymal transition (EndMT) [73]. Additionally, ICPs are involved in anti-apoptosis [74], angiogenesis [75], and enhanced energy metabolisms [76,77].

As membrane proteins, ICPs are translocated to the cell membrane via exocytic secretory pathways. As we reported, canonical secretomes [51,52,59,78] are initially processed in the endoplasmic reticulum (ER) [55, 72] and then delivered to the cell surface to exert their inhibitory functions. This process involves the sequential transportation of secretomes, which may include ICPs, through the Golgi apparatus and secretory vesicles, facilitated by the protein-sorting system. During cell surface delivery, several regulatory metabolic mechanisms are involved: (a) glycosylation acts as a quality control [79, 80] measure to ensure that only mature and functionally modified ICPs are delivered to the surface, as we previously reported for other membrane proteins [80]; (b) internalization and recycling [81] occur once the ICPs reach the cell surface, providing a rapid regulatory pathway to adjust their surface levels; (c) ubiquitination-mediated protein degradation is another crucial mechanism to control ICP expression on the membrane. ICPs can be ubiquitinated and directed to the proteasome or lysosome for degradation [2]. These cellular processes together determine the surface level of ICPs, thereby shaping cell signaling [82].

Metabolic reprogramming has been found in immune cells. Immune cells adapt their metabolic pathways to perform a variety of functions, opening up numerous possibilities for interventions aimed at regulating inflammation. This adaptability is largely achieved by shifting their use of substrates towards mitochondrial oxidative phosphorylation (OXPHOS), glycolysis, or other pathways like the pentose phosphate pathway. Such metabolic choices significantly influence cellular development, fate, and behavior. Similar to the metabolic reprogramming associated with innate immune memory (trained immunity) in innate immune cells, which we have reviewed [54,56,83] and reported [18,55,59,84–87], glycolysis plays a crucial role in metabolic reprogramming and immunoregulation. Consequently, numerous therapies targeting glycolysis have been developed, and their combinations with immune checkpoint inhibitors (ICPIs) in preclinical and clinical trials suggests that ICP signaling pathways interact with metabolic reprogramming. Additionally, increasing attention is being given to the roles of amino acids, lipids, nucleotides, and mitochondrial biogenesis in metabolic rewiring regulated by ICPs in immune cells [88]. The diverse functions of ICPs and their tissue expression profiles may be significant for CVDs and cerebrovascular diseases. Additionally, antigen-specific CD8+ T cells undergo metabolic reprogramming via IRF4 to sustain a high glycolytic rate, which is essential for their clonal expansion and effector function [89]. Additionally, metabolic selection plays a crucial role in T cell differentiation; glycolysis promotes the development of often pathogenic TH17 cells, while inhibiting HIF1α, a glycolysis regulator, favors the generation of FOXP3+ regulatory T (Treg) cells [90]. In adipose tissue, Treg cells are vital for maintaining immune balance and suppressing inflammation, making them a promising target for immunometabolic therapies.

## 3. Regulation of Innate Immune Cells, Tregs, and T Cells by Immune Checkpoints in Cardiovascular Diseases Pathogenesis

Immune checkpoints play a crucial role in regulating various aspects of immune function, and recent research highlights their significant impact on CVD pathogenesis. Fourteen pairs of immune checkpoints have been identified as key regulators of innate immune cells, Tregs, and conventional T cells (Table 2). These checkpoints modulate the balance between immune activation and suppression, influencing inflammation and immune responses within the cardiovascular system. By understanding how these immune checkpoints affect CVDs, researchers aim to develop targeted therapies that can effectively manage or prevent these conditions. Several preclinical studies have demonstrated that targeting immune-checkpoint pathways can be beneficial in reducing atherosclerotic plaque formation, inflammation in adipose tissue, and insulin sensitivity [91,92]. For instance, CTLA-4 and PD-1, along with their ligands, play roles in suppressing cardiovascular inflammation and promoting CD4^+^Foxp3^+^ Treg-mediated immunosuppression. CTLA-4 inhibits T-cell activation by competing with CD28 and downregulating the expression of the ligands CD80 and CD86. Interestingly, deficiencies in CD80 and CD86 molecules can prevent the progression of atherosclerosis in low-density lipoprotein receptor knockout (*LDLR)*^−/−^ mice by suppressing proatherogenic type 1 T helper cell (Th1) immune responses [37]. Conversely, reconstitution with *CD80*^−/−^*/CD86*^−/−^ or *CD28*^−/−^ bone marrow in *LDLR*^−/−^ mice inhibited Treg development and exacerbated atherosclerosis, indicating that the CD80/CD86-CTLA4 axis in bone marrow-derived cell types is crucial for Treg development and Treg suppression of atherosclerosis. In contrast, CTLA-4 transgenic (*CTLA-4-Tg)/apolipoprotein E (ApoE)*^−/−^ *mice* showed markedly reduced atherosclerotic lesion formation, less intraplaque accumulation of macrophages and CD4^+^ T cells in the aortic root, and decreased expression of CD80 and CD86 on CD11c^+^ DCs, affecting reverse signaling [38]. Treatment with soluble agonist CTLA-4 Ig fusion protein reduced atherosclerosis progression in hyperlipidemic and hyperhomocysteinemic *ApoE*^−/−^ mice by competitively inhibiting CD28-CD80/86 co-stimulatory T-cell activation and pro-inflammatory cytokine release [93,94]. CTLA-4, which constitutively expressed on CD4^+^Foxp3^+^ Tregs, is crucial for Treg-mediated suppression [95]. CTLA-4 and Tregs cooperate through complementary and largely overlapping mechanisms to maintain immune tolerance. Tregs typically use CTLA-4 to carry out their suppressive effects. However, CTLA-4 can also act in non-Treg cell types. Additionally, Tregs have the ability to suppress immune responses through CTLA-4-independent mechanisms [96]. In *CTLA-4-Tg/ApoE*^−/−^ mice, although there is no significant change in the proportion of Foxp3^+^ Tregs in the aortic root plaques, CTLA-4 overexpression enhances the suppression efficacy of CD4^+^CD25^high^ Tregs on CD4^+^CD25^−^ T effector cell proliferation and activation. Additionally, CTLA-4 overexpression also increases Tregs’ inhibition of DC maturation [38]. Tregs not only downregulate CD80/CD86 expression on DCs and macrophages [23], but they also reduce CD86 expression on ECs (innate immune cell type as we reported [56,58,86,97–99]) when these cells are stimulated by oxidized low-density lipoprotein (ox-LDL) and lipopolysaccharide (LPS) [100]. Blockage of CTLA-4 disrupts Treg-mediated suppression of EC activation, leading to increased expression of inflammatory cytokines and adhesion molecules [101].

Another well-known co-inhibitory immune checkpoint [51], PD-1, plays a significantly different role from CTLA-4 in suppressing immune responses. While CTLA-4 is thought to regulate T-cell proliferation early in the immune response, mainly within lymph nodes, PD-1 acts later, primarily suppressing T cells in peripheral tissues [102]. PD-1 is induced on T cells, B cells, macrophages, and certain DCs [103]. PD-1 binds to its ligands, PD-L1 and PD-L2 [104]. PD-L1 is expressed on a broad range of cell types, including both hematopoietic and non-hematopoietic cells. While, PD-L2 expression is primarily restricted to DCs and macrophages. In hypercholesterolemic mice, genetic knockout of PD-1 or treatment with PD-1 antibodies did not affect lesion size after 5-week of high-fat diet (HFD) feeding but resulted in a significant increase in inflammatory cells, including CD4^+^ and CD8^+^ T cells and macrophages. After 10 weeks on the HFD feeding, *PD1*^−/−^*/LDLR*^−/−^ mice showed increased lesion size and a greater accumulation of CD4^+^ and CD8^+^ T cells and macrophages, compared to *LDLR*^−/−^ controls [39, 105]. PD-1 deficiency did not affect VSMC content (also innate immune cells as we reported [59,72,106]) but led to enhanced CD8^+^ cytotoxicity and increased VSMC death within the lesions [39]. These findings from gene-deficiency CVD models suggest that ICPs play molecule-specific, cell-type-specific, and disease-stage-specific roles in CVD progression.

Genetic knockout of PD-L1/2 in *LDLR*^−/−^ mice led to increased atherosclerotic lesion formation, accompanied by a rise in lesional CD4^+^ and CD8^+^ T-cells, elevated serum levels of tumor necrosis factor-a (TNF-α), and more potent APCs in activating CD4^+^ T cells [40]. Consistently, in *PD-L1/2*^−/−^ bone marrow transplantation into *LDLR*^−/−^ and control *PD-L1/2*^+/+^ bone marrow transplantation into *LDLR*^−/−^, lesions of *PD-L1/2*^−/−^ marrow recipients showed increased numbers of CD4^+^ T cells, CD8^+^ T cells, and macrophages. These findings suggest that PD-L1 and PD-L2 expression on bone marrow-derived cells plays a crucial role in suppressing proatherogenic T-cell responses. Notably, the lesion size was comparable between the two recipient groups, which could be due to the necessity of PD-L1 deficiency on both non-hematopoietic and hematopoietic cells (APCs) to affect lesion size. Additionally, the bone marrow transplantation procedure, involves sub-lethal irradiation, might impact the function of mature immune cells [107,108]. The balance of PD-1 expression between Teffs and Tregs can predict the clinical efficacy of PD-1 blockade therapies [109]. PD-1 blockade significantly enhances the suppressive activity of Tregs in vitro. In mouse models, genetic ablation or antibody-mediated blockade of PD-1 in Tregs results in increased Treg proliferation and enhanced suppression of immune responses [110]. Although there was no significant difference in the numbers of CD4^+^Foxp3^+^ Tregs between *PD1*^−/−^/*LDLR*^−/−^ and *LDLR*^−/−^ mice at either 5 or 10 weeks [39], a notable increase in naïve and atheroprotective CD4^+^Foxp3^+^CD25^high^ Treg subsets [111] was observed in circulating blood, spleen, and aortic root of *PD1*^−/−^/*LDLR*^−/−^ mice. These Tregs maintained normal suppressive function in vitro [105]. These findings collectively indicate that PD-1 expressed on effector Tregs may act as a negative regulator of Treg cell-mediated immunosuppressive function and Treg cell proliferation. Furthermore, the effect of PD-L1/2 deficiency is primarily mediated through dysregulated T cell activation by APCs [40]. Another possible reason for enhanced proatherogenic T cell responses could be impaired Treg development or function [111,112]. PD-L1 plays a pivotal role in regulating the development and function of induced Treg cells (iTregs) [113]. However, there is no clear evidence indicating that PD-L1 or PD-L2 is involved in the suppression function of natural (thymic) Tregs [50,114]. Naive Tregs isolated from PD-L1/2–deficient mice also did not show impairment in their suppressive function [40]. These observations from gene-deficiency models in CVD suggest that ICPs and their ligands have specific roles depending on the cell-types involved.

Vascular inflammation driven by T-cell-mediated immune responses is critically involved in the pathogenesis of abdominal aortic aneurysm (AAA). Modulating the balance between Tregs and effector T (Teff) cells to favor Treg functions could be a promising therapeutic approach for preventing AAA formation [115,116]. CTLA-4 binds to CD80 and CD86, leading to trogocytosis (a biological process where one cell “nibbles” another cell, transferring surface molecules and membrane fragments between them), where one cell physically extracts and ingests portions of materials from another cell. This process results in the release of free PD-L1 from APCs, disrupting the cis-CD80/PD-L1 heterodimers on APCs. Consequently, there is increased interaction between PD-1 and PD-L1 on T cells [117,118], leading to the negative regulation of T cell function [119]. CTLA-4 overexpression in T cells effectively prevents AAA formation in angiotensin II-infused hypercholesterolemic mice [72,120]. In contrast, immunohistochemical analysis of human AAA samples revealed co-expression of PD-1 with CD3 (a T cell marker), CD68 (a macrophage marker), and α-actin (a VSMC marker). Treatment with PD-1 antibodies or BMS-1 (a PD-1 inhibitor) significantly inhibited AAA progression in both the mouse CaCl_2_ incubation model and the rat pseudoaneurysm progression model. This treatment reduced macrophages, lymphocytes, and apoptosis of VSMCs, as wells as vessel wall calcification [121]. These findings demonstrate that, despite both CTLA-4 and PD-1 being co-inhibitory receptors, their role in CVDs leads to different outcomes.

T cell immunoreceptor with Ig and ITIM domain (TIGIT) exerts diverse immunosuppressive effects on the cancer immunity cycle, including inhibiting NK cell effector functions, suppressing DC maturation, promoting macrophage polarization to the M2 phenotype, and facilitating the differentiation of T cells into Tregs [122–124]. TIGIT predominantly suppresses immune responses and inflammation through its influence on Tregs [125,126]. TIGIT^+^ Tregs selectively inhibit proinflammatory Th1 and Th17 cell responses. Additionally, TIGIT signaling restores the suppressor function of Th1 Tregs (also referred to as plastic Tregs or pathological Tregs, as we previously defined [50]) [127,128]. The high fat diet feeding (HFD) induces TIGIT expression in CD4^+^ T cells, particularly in Tregs with highly activated phenotypes, which include enriched migratory molecules and increased expression of immunosuppressive genes [45,129]. Meanwhile, atherosclerosis-driven dysfunctional/plastic interferon- γ (IFNγ) ^+^C-C motif chemokine receptor 5 (CCR5) ^+^ Th1-Tregs in ApoE^−/−^ mice show reduced TIGIT expression [130], suggesting that TIGIT plays a stabilizing role in Tregs, helping to suppress inflammation [131,132]. A gain-of-function model using agonistic anti-TIGIT treatment inhibited T cell responses in *LDLR*^−/−^ mice [129] but did not affect early atherosclerosis development during 4 and 8 weeks of a HFD feeding. The authors suggest that this might be due to the agonistic anti-TIGIT blocking the natural interaction between TIGIT and its ligand CD155, resulting in increased percentages and activation of pro-inflammatory DCs [125,133]. To better understand these mechanisms, the use of global and conditional TIGIT-deficient mice is recommended for future studies.

TIGIT binds to CD155 on APCs [131] to impair T cell priming [124]. It competes with co-stimulatory receptors CD226 and CD96 on T cells for binding to CD155, with TIGIT having a higher binding affinity than its competitors [134]. Similar to the CD28/CTLA-4 pathway, TIGIT and CD96 act as co-inhibitory receptors, contrasting with the co-stimulatory receptor CD226. In addition to CD155 (PVR), TIGIT also binds to three other ligands on APC membrane: CD112, CD113, and PVRL4 (nectin cell adhesion molecule 4, NECTIN4), forming a complex regulatory axis [135,136]. A recent study found that CD155 is highly expressed on macrophages in patients with coronary artery disease (CAD), delivering negative signals to CD4^+^ T cells expressing CD96 and/or TIGIT receptors [137]. This combination of increased inflammatory potential and reduced antigen-presenting function appears unique to CAD macrophages [138]. The hyperlipidemia induced TIGIT-CD155 axis reflects this excess inflammation, coupled with impaired APC function and T cell response [129]. TIGIT^+^ Tregs upregulate the expression of the co-inhibitory receptor TIM-3, creating a synergistic effect that further suppresses immune responses [125].

Hepatitis A virus cellular receptor 2 (TIM-3, *Havcr2*) is an emerging co-inhibitory checkpoint originally identified as a receptor on interferon (IFN) - γ -producing CD4^+^ and CD8^+^ T cells [139]. However, in the diseased aorta, TIM-3 is consistently found on myeloid cells, such as monocytes and macrophages, rather than on T cells. TIM-3 plays a crucial role in the phagocytosis of apoptotic cells and cross-presentation, potentially contributing to peripheral tolerance through its interactions with GAL-9, encoded by LGLAS9, which is expressed on peritoneal exudate macrophages, monocytes, and splenic DCs [140]. GAL-9 is expressed by macrophage subsets in progressing plaques, and in vitro studies show that exogenous GAL-9 promotes a pro-inflammatory phenotype in macrophages [141]. *GAL-9*^−/−^*/ApoE*^−/−^ mice on a HFD exhibit significantly reduced aortic plaque burden without changes in circulating leukocyte subset levels. In contrast, hypercholesterolemic *LDLR*^−/−^ mice treated with anti-TIM3 show increased atherosclerotic plaque formation, along with elevated levels of monocytes and lesional macrophages, an increase in CD4^+^ T cells, and a reduction in Tregs and regulatory B cells (Bregs) [142]. These findings suggest that the GAL-9/TIM-3 axis plays a crucial role in multiple cell-cell interactions within atherosclerotic lesions, with TIM-3 activation suppressing GAL-9-driven inflammation.

Additionally, TIM-3 binds to the nuclear protein high mobility group box protein 1 (HMGB1), a damage-associated molecular pattern (DAMP)/alarmin [143], and negatively regulates HMGB1-mediated activation of the innate immune response to nucleic acids [144]. HMGBP1, typically found in the nucleus with DNA-binding capabilities, can be released from damaged cells to perform extracellular functions [145]. Lung-derived HMGB1 can be captured by arterial macrophages, altering their mitochondrial metabolism [18, 146], elevating oxidative stress [147 – 151], and triggering arterial matrix degradation, thereby promoting AAA formation [152]. While macrophages play a key role in AAA formation through HMGB1 production [153], single-cell RNA sequencing (scRNA-seq) revealed that HMGB1 is highly enriched in adipocyte, neurons, mesothelial cells (form a thin protective layer called “mesothelium”, covering the whole serous cavities and the entire surface of internal organs), and VSMC within the AAA aorta [154]. This suggests a potential mechanism through which the TIM-3/HMGB1 axis may inhibit AAA progression. In addition to the co-expression of TIGIT and TIM-3 on Tregs, studies have also shown that the co-expression of PD-1 and TIM-3 on CD8^+^ T cells is upregulated in patients with atherosclerosis [155]. PD-1^+^TIM-3^+^CD8^+^ T cells in freshly isolated peripheral blood mononuclear cells (PBMCs) from local lesional arteries display an anti-atherogenic function. This co-expression is associated with reduced levels of pro-atherogenic cytokines (IFN-γ and TNF-α) and increased levels of anti-atherogenic cytokines (IL-10 and IL-4) compared to CD8^+^ T cells expressing only one of these receptors. Dual blockade of PD-1 and TIM-3 reduces IL-10 and IL-4 production in CD8^+^ T cells, with stronger effect compared to treatment with either anti-TIM-3 or anti-PD-L1 alone. Meanwhile, treatment with both anti-TIM-3 and anti-PD-L1 antibodies increases INF-γ and TNF-α levels. These findings provide additional insights into the potential risks of atherosclerosis, especially considering that PD-1 and Tim-3 are targets of emerging therapies aimed at treating viral infections [156,157].

Lymphocyte activating 3 (LAG-3, CD223) is another recognized ICP that delivers inhibitory signals, regulating immune cell homeostasis, T cell activation and proliferation, cytokine production, and cytolytic activity [158]. In studies evaluating LAG-3′s role in atherosclerosis, mice with *LAG-3*^−/−^ bone marrow transplantation to *LDLR*^−/−^ or those treated with blocking anti-LAG-3 monoclonal antibodies showed increased levels of interferon-g (IFN-γ)–producing Th1 cells and effector/memory T cells [159]. However, this increase in IFN-γ–producing Th1 cells and effector/memory T cells was balanced by higher levels of Tregs, and neither condition affected atherosclerotic plaque size. Additionally, treatment with anti-LAG-3 in HFD fed ApoE^−/−^ mice led to enhanced PD-1 expression in both CD4^+^ and CD8^+^ T cells and induced IL-2 production in IFN- γ -producing CD8^+^ T cells, suggesting that anti-LAG-3 antibodies may have an immunostimulatory effect in suppressing cardiovascular inflammations similar to anti-PD-1 therapy. The LAG-3 ligand, Galectin 3 (GAL-3, encoded by Lgals3), has been identified as a key cytokine regulation hub that mediates immunological functions across various immune cells, including DCs, B cells, and macrophages. Inhibition of GAL-3 downregulates the expression of pro-inflammatory cytokines such as IL-6, IL-1β, and IL-23p19, while simultaneously upregulating anti-inflammatory cytokines like IL-10 and IL-12p35 [160,161]. Additionally, GAL-3 is highly expressed in macrophages within human atherosclerotic plaques and in murine models of atheroma [162,163]. GAL-3 is chemotactic for macrophages, serving as a stimulus for monocyte recruitment during plaque progression and reducing M2 activation of plaque macrophages [164]. Studies have shown that GAL-3 deficiency leads to a 50% reduction in middle and later atherosclerotic lesions after 12–20 weeks of HFD feeding.

GAL-3 has been shown to be elevated in heart failure models [165] and has prognostic value in heart failure patients, being implicated in cardiac fibrosis and remodeling [166,167]. Additionally, high circulating GAL-3 levels predict major adverse clinical outcomes following acute myocardial infarction [168] and all-cause mortality in the general population [169]. GAL-3 levels are also elevated in the plasma of AAA patients compared to controls and are associated with the need for surgical repair, independent of potential confounding factors [170]. Modified citrus pectin (MCP), a GAL-3 inhibitor, has been shown to reduce aortic dilation in mice while also decreasing elastin degradation, VSMC loss, and macrophage content. These findings underscore the potential role of GAL-3 as a therapeutic target in AAA [171].

The cytotoxic activity of myeloid cells is regulated by a balance between signals transmitted through inhibitory and stimulatory receptors. CD47 plays a pivotal role in this balance by delivering a “don’t eat me signal” in both healthy and diseased cells [172,173]. Signal regulatory protein alpha (SIRPα), a receptor found on myeloid cells, binds to CD47 with high affinity [174], contributing to resistance against phagocyte-dependent clearance [175]. In advanced atherosclerotic arteries, impaired efferocytosis—the process by which apoptotic cells are cleared by both professional and non-professional phagocytes – exacerbates lesion progression and plaque necrosis [176,177]. Anti-CD47 treatment reduces vascular inflammation in human patients and ameliorates atherosclerosis in hypercholesterolemic ApoE^−/−^ mice by correcting defects in efferocytosis and normalizing the clearance of diseased vascular tissue, leading to the regression of atherosclerosis [178,179]. CD47^−/−^/ApoE^−/−^ mice are protected from atherosclerosis, and systemic inhibition of SIRPα -mediated signaling appears more effective in reducing the necrotic core area in murine atherosclerosis models compared to global CD47 inhibition [180]. Furthermore, CD47 and SIRPα expression are elevated in human atherosclerotic arteries, with both primarily co-localized in areas of CD68^+^ macrophages within the plaque region. Deficiency in myeloid cell-specific SIRPα enhances efferocytosis, reduces cholesterol accumulation, promotes lipid efflux and attenuates oxidized LDL-induced inflammation in vitro. Additionally, it induces the M2 macrophage phenotype, inhibits necrotic core formation in the arterial wall in vivo, reduces inflammation, and suppresses atherosclerosis. Conversely, the loss of CD47 on myeloid cells decreases macrophages’ cholesterol efflux and promotes NF-κβ nuclear translocation, leading to increased atherosclerotic lesion formation. These findings highlight SIRPα as a potential therapeutic target in atherosclerosis and emphasize the need for further research into the cell-specific role of CD47 in the arterial wall.

*Selectin P ligand (Selplg)* encodes PSGL-1, a high-affinity counter-receptor for P-selectin found on myeloid cells and activated T cells. Numerous studies have highlighted the essential role of P-selectin and PSGL-1 in the formation of atherosclerotic lesions, thrombosis, and alterations in arterial walls [181,182]. V-set immunoregulatory receptor (VISTA, VSIR), a novel member of the mouse immunoglobulin superfamily, is constitutively expressed on CD11b^+^ myeloid DCs, naive CD4^+^ and CD8^+^ T cells, and CD4^+^Foxp3^+^ regulatory T cells. Similar to CTLA-4 and PD-1, VISTA plays a role in regulating peripheral tolerance and immunity [183]. As an acidic pH-selective ligand for PSGL-1, VISTA engages and suppresses T cells selectively in acidic environments [184]. The PH within atherosclerotic lesions has been demonstrated to vary between 6.5 and 8.5, reflecting both individual variations and the complex nature of plaque pathology [185]. Recent research using the pH-sensitive pHrodo probe—an intracellular pH indicator dyes that fluoresces under acidic conditions (pH < 7) —has identified acidic regions within atherosclerotic lesions, which are associated with macrophages, IgE, and cell apoptosis [186]. Both Selplg and VISTA are significantly upregulated in mouse AAA and atherosclerotic lesions, with their cellular localizations being enriched in T cells and macrophages, respectively, under these conditions. While the VISTA in AAA and atherosclerosis has not been fully reported, *VISTA*^−/−^ mice show spontaneous immune-cell infiltration in the lung, liver, and pancreas, along with activation of IL-23/IL-17-mediated inflammatory responses and impaired function of Foxp3^+^CD4^+^ Tregs [187, 188]. These observations suggest that the Selplg/VISTA axis may play a role in counteracting AAA and atherosclerotic progression through its immunosuppressive functions.

CD40/CD40L signaling is essential for the development of CVD treatments [189]. Our research has shown that chronic kidney disease (CKD) induces the differentiation of inflammatory CD40^+^ monocytes through elevated homocysteine levels and DNA methylation [68,190,191]. Similar to other pro-inflammatory cytokines, soluble CD40 ligand (sCD40L) is recognized as a cytokine-like molecule and an independent cardiovascular risk factor [192, 193]. Anti-CD40L antibody therapy not only limits early atherosclerosis in *LDLR*^−/−^ mice, significantly reducing macrophages and T cells [194], but also inhibits the progression of established atherosclerotic lesions [195]. However, studies using *CD40L*^−/−^*/ApoE*^−/−^ mice revealed that significant differences were observed only in advanced plaques, not in initial plaques, compared to ApoE^−/−^ controls [196]. This finding aligns with our recent report that the cytokine secretome in the early aorta of ApoE^−/−^ mice (after 6 weeks of HFD) differed from that in advanced atherosclerotic aorta (32–78 weeks of HFD feeding) [106]. This suggests that CD40-CD40L signaling plays a crucial role in late atherosclerotic changes, such as lipid core formation and plaque destabilization [197]. Unlike the well-established role of CD40 expressed on APCs, it remains unclear which CD40L expressing cell type(s) are responsible for the various aspects of atherogenesis [198,199]. A recent study investigated the contributions of T cell- and platelet-specific CD40L, the two primary expression cell types of CD40L [200], in atherosclerosis using conditional gene-deficient models [108]. The study demonstrated that the absence of T cell CD40L reduces plaque burden, resulting in less advanced and more stable plaques, whereas the absence of platelet CD40L does not affect atherosclerotic plaque progression but reduces platelet deposition and suppresses thrombus formation. Additionally, CD40-deficiency in CD11c^+^ DCs leads to decreased atherosclerosis, accompanied by a reduction in plaque T cells and a systemic reduction in Tregs. These findings highlight the critical roles of the T cell-, Treg-, and DC CD40L-CD40 axis in atherogenesis.

The co-stimulatory molecule CD70, expressed on activated immune cells, modulates T, B, and NK cells via its receptor CD27 [201]. Studies have shown that CD70 expression is elevated in ruptured human carotid atherosclerotic plaques compared to stable plaques, with CD70 predominantly localized to macrophages in murine atheroma [202]. The absence of CD70 impairs the inflammatory capacity of bone marrow-derived macrophages, specifically reducing their production of reactive oxygen species (ROS) and nitric oxide (NO). This deficiency also increases both M1-like and M2-like macrophage markers, rendering the macrophages metabolically inactive and more susceptible to apoptosis, highlighting the critical role of CD70 in maintaining macrophage function and survival in inflammatory environments. CD70-deficient macrophages show reduced expression of scavenger receptors and ATP-binding cassette (ABC) -transporters, leading to impaired uptake of oxLDL and decreased cholesterol efflux. Despite these deficiencies, *CD70*^−/−^ *bone marrow transplantation into ApoE*^−/−^ [13,53] exhibit a significant increase in necrotic core size, plaque area, and lesion macrophages compared to controls [202], suggesting that CD70-deficient macrophages are less capable of clearing the lipid deposits from the arterial wall and promoting the progression of atherosclerotic lesions. These findings underscore CD70′s role in mitigating atherosclerosis by regulating macrophage’s function.

On the other hand, CD27 is expressed on naïve T cells under steady state conditions and is also found on NK cells, activated B cells, and hematopoietic stem cells in mice. CD27 promotes the development of Tregs through its interactions with CD70 on ECs and DCs in the thymic medulla [203]. ApoE^−/−^ mice reconstituted with CD27-deficient bone marrow show significantly larger plaque sizes and a decreased abundance of Tregs compared to mice receiving *CD27*^+/+^*ApoE*^−/−^ bone marrow, particularly in the early stages of atherosclerosis [204]. Notably, adoptive transfer of wild-type Tregs into CD27-deficient mice reversed this phenotype, resulting in even smaller lesions than those in control mice, suggesting that CD27^+^ Tregs play a protective role against atherosclerosis. Additionally, the study reveals that peripheral Tregs lacking CD27 show similar suppressive capacities compared with their wild-type counterparts. However, thymic CD27^−/−^ Tregs exhibit increased apoptosis and express fewer proliferation markers *in vivo*, indicating that CD27 deficiency impairs thymic output of Tregs, contributing to the exacerbation of atherosclerosis, particularly in the initial stages.

As previously discussed, T cells play a distinctive role in arteriogenesis and angiogenesis. However, the specific role of co-stimulation in T cell activation during neovascularization remains to be fully established. In hindlimb ischemia models, blood flow recovery was compared between CD70^−/−^, CD80^−/−^/86^−/−^, CD70^−/−^/80^−/−^/86^−/−^, and CTLA4^+/−^ mice [201]. The results showed that blood flow recovery was significantly impaired in mice lacking CD70, with a reduced number of pre-existing collaterals compared to control mice. In contrast, blood flow recovery was similar in CD80^−/−^/86^−/−^, CTLA4^+/−^, and control mice. Additionally, CD70 deficiency impairs VSMCs from activating T cells, highlighting the CD27-CD70 axis as a crucial co-stimulation pathway for pre-existing collateral formation and post-ischemic blood flow recovery, playing a key role in arteriogenesis and angiogenesis.

The deficiency of TNF superfamily member 14 (Tnfsf14, which encodes LIGHT), leads to an increased maximum diameter of the abdominal aorta aneurysm and heightened severity of AAA, alongside a loss of the VSMC contractile phenotype. These findings suggest that TNFSF14 play a protective role by preventing VSMC trans-differentiation [60,106,205]. Although TNFRSF14/TNFSF14 expression does not show consistently or significantly change in atherosclerosis, in vitro studies reveal high levels of TNFRSF14 expression in activated monocytes, macrophages, and THP-1 cells (a monocyte cell line) when induced by proinflammatory cytokines and matrix metalloproteinases [206]. In addition to interacting with canonical TNF-related ligands such as TNFSF14 and lymphotoxin a (LT-α), TNFRSF14 also binds to immunoglobulin superfamily members like B and T lymphocyte attenuator (BTLA) and CD160, as well as the herpes simplex virus (HSV) glycoprotein D [207,208]. This broad range of ligand interaction enables TNFRSF14 to activate various intrinsic and bidirectional signaling pathways. Notably, the expression of CD160 is significantly reduced in the mouse aorta as atherosclerosis progress [209]. Unlike its lower affinity binding to MHC-I, which enhances NK cell and CD8^+^ cytotoxic functions, CD160 binds to TNFRSF14 with higher affinity, leading to the inhibition of T cell responses [210]. In patients with atherosclerosis, CD160 is significantly upregulated on peripheral NK cells; however, this effect may be offset by a concurrent decrease in the number of peripheral NK cells [211]. In CKD patients, the downregulation of BTLA was linked to the development of de novo plaques presence after 2 years. However, this downregulation was not associated with further atherosclerotic progression in patients who already had plaques at baseline [212]. In addition, BTLA expression was significantly reduced in both blood samples and atheroma plaques from CKD-accelerated atherosclerosis mouse models [212], as well as in macrophages treated with uremic serum. These findings suggests that BTLA could serve as a potential biomarker or therapeutic target for atherosclerosis in CKD patients.

Glucocorticoid-induced tumor necrosis factor receptor family-related protein (GITR), encoded by Tnfrsf18, is primarily expressed on CD4^+^ Teffs, NK cells, and Tregs. It is activated by its ligand, GITRL, which is mainly found on APCs and ECs [213]. In a study using B cell-restricted GITRL transgenic mice reconstituted with LDLR^−/−^ mice, systemic expansion of Tregs over Teffs was observed with continuous GITR stimulation, resulting in significantly less severe atherosclerosis [214]. In contrast, a recent study by the same research team identified that GITR drives atherosclerosis in mice and is associated with an unstable plaque phenotype and cerebrovascular events [24] in humans [215]. GITR expression was elevated in carotid endarterectomy (a surgical procedure that removes plaque from the carotid arteries to reduce the risk of stroke) specimens from patients with cerebrovascular events compared to asymptomatic patients. This elevated expression correlated with indicators of plaque vulnerability, including plaque macrophages, lipid content, glycophorin A (GPA), and levels of pro-inflammatory cytokines such as IL-6, IL-12, and C-C motif chemokine ligand 2 (CCL-2). Soluble GITR levels are elevated in the plasma of individuals with CVD compared to healthy controls. Genetically deficient *GITR*^−/−^*/ApoE*^−/−^ mice exhibit reduced plaques with fewer macrophages, a smaller necrotic core, and a thicker fibrous cap compared to controls, although lymphoid cell populations remain unchanged. RNA sequencing of monocytes and macrophages revealed that GITR is not only expressed in T cells but also in macrophages, VSMCs, and ECs of the arterial wall. These findings indicate that GITR expression in monocytes and macrophages plays a critical role in driving atherosclerosis, potentially outweighing its effect in T cells.

TNF Receptor Superfamily Member 4 (OX40, encoded by Tnfrsf4) and OX40L (encoded by Tnfsf4) are crucial components of the TNF/TNFR family, forming a key co-stimulatory axis. The interaction between OX40, expressed on T cells, and OX40L, found on APCs and ECs, plays a vital role in T cell proliferation and survival, particularly promoting Th2 responses [49, 216]. Studies have shown that OX40L deficiency reduces susceptibility to atherosclerosis, while OX40L overexpression accelerates the development of the disease [217]. Blocking the OX40-OX40L interaction with OX40L antibodies leads to a reduction in the initiation of atherosclerosis [218]. Furthermore, administering anti-OX40L treatment after 10-weeks on HFD in *LDLR*^−/−^ mice results in significant regression of aortic lesions, reduced OX40 expression, and fewer T cells in the adventitia, without altering the phenotype of plaque macrophages. This treatment also induces IL-5 producing T cells in the peritoneum, promoting athero-protective oxLDL-specific IgM secretion through an IL-33-dependent pathway [219, 220]. Beyond affecting T cell dependent humoral responses, inhibiting the OX40-OX40L interaction also diminishes Th2 responses and inhibits mast cell function, thereby reducing mast cell-mediated T cell proliferation and cytokine production [221].

## 4. Discussion

In summary, our review highlights the critical roles that ICPs play in the regulation of a wide range of CVDs. Beyond the initial CVDs discussed—such as vein graft disease [35], graft arterial disease [36], atherosclerosis [37 – 40], coronary heart disease and ischemic stroke [25,26], myocarditis [41], cardiomyopathy [42], and heart failure [43]—ICPs are also significantly involved in the pathogenesis of additional 17 CVDs. These include hyperlipidemia-induced vascular inflammation, hyperhomocysteinemia [222]-associated vascular inflammation [93, 94], AAA [72, 120], pseudoaneurysm progression [121], atherosclerosis regression and efferocytosis [178, 179], acidic atherosclerotic lesions associated with macrophages, IgE and cell apoptosis [186], late atherosclerotic changes with lipid core formation and plaque destabilization [197], cardiac fibrosis, remodeling and heart failure [165–167], acute myocardial infarction [168], CKD-accelerated CVD [68, 190, 191], thrombus build-up [108,200], early atherosclerosis [204] and initiation of atherosclerosis [218], VSMC trans-differentiation [205], and an unstable plaque phenotype associated with cerebrovascular events in humans [215].

Based on the studies discussed, we propose the following immunological principles to guide the future development of new ICP therapeutics for CVDs: (1) *Disease stage-specific expression*: ICP blockade therapy should consider the disease stage specific expression of ICPs, as observed in conditions such as graft arterial disease [46,48] and atherosclerosis [196]. Tailoring treatments to the disease stage could enhance therapeutic efficacy. (2) *Distinct cell type focus*: Different ICPs target specific cell types. For example, CTLA-4 primarily regulates early T-cell proliferation in lymph nodes, while PD-1 acts later by suppressing T cells mainly in peripheral tissues [102]. To fully understand the roles of various ICPs in different cell types and overall global CVD pathologies, employing both conditional knockout (KO) and global KO models of ICPs is essential. (3) *Cellular functions of ICPs*: ICPs initially identified in anti-tumor immunotherapies, also plays roles in CVD pathologies and cerebrovascular diseases including: (a) self-renewal: regulation of self-renewal in CVD-related stem cells and progenitors [69], (b) epithelial-mesenchymal transition (EMT) [70]: similar to the phenotype switch [71] and trans-differentiation [72] of VSMCs and EndMT [73], (c) metastasis [223], (d) drug resistance [224], (e) anti-apoptosis [74], (f) angiogenesis [75], and (g) enhanced energy metabolisms [76, 77]. (4) *Expression balance of ICPs*: The balance of ICP expression, such as PD-1, between proinflammatory/pro-immune effector T cells and anti-inflammatory/immunosuppressive Tregs, can predict the clinical efficacy of ICP blockade therapies [109]. Maintaining this balance is crucial for successful treatment outcomes. (5) *Therapies targeting co-stimulatory receptors*: Given the distinct roles of T cells in arteriogenesis and angiogenesis, co-stimulatory receptors, such as CD27-CD70 axis, offer new therapeutic potential for enhancing post-ischemic blood flow recovery. (6) *Antibody treatment considerations*: Antibody treatments can be either antagonistic or agonistic. However, agonistic antibodies may inadvertently block receptor-ligand interactions, leading to unintended outcomes [129]. This potential complication must be carefully considered during therapy development. (7) *Cell type specific expression*: ICPs exhibit cell type-specific expression patterns and functional redundancies, as demonstrated by the varied ICP expression across different human tissues [22]. This immune heterogeneity underscores the complex roles of ICPs and their ligands in atherogenesis and CVDs, which can vary depending on the cell type or the organ involved. When developing ICP-based therapies, its crucial to differentiate between antibody treatments and gene deletions. antibodies block extracellular interactions, potentially leaving intracellular signaling pathways intact [225]. Therefore, understanding whether ICPs predominantly affect effector T cells or Tregs can guide the development of targeted ICP-based therapeutics for CVDs and cerebrovascular diseases.

Mapping the landscape of disease stages and cell types regulated by immune checkpoint axes in the context of CVD could help identify potential therapeutic targets. This approach would also be crucial for monitoring the safety of applying immunotherapies—originally designed for cancer and chronic infections—to patients with CVDs.

## Figures and Tables

**Table 1.** Immune checkpoint couples (60 pairs).

APC	T-cell	Category	APC	T-cell	Category
CD40	CD40LG	Stimulatory	HLA-G	KIR2DL4	Inhibitory
CD48	CD2	Stimulatory	HMGB1	HAVCR2	Inhibitory
CD58	CD2	Stimulatory	IGSF11	VSIR	Inhibitory
CD70	CD27	Stimulatory	ITGA2	CD49B	Inhibitory
CD80	CD28	Stimulatory	LGALS3	LAG3	Inhibitory
CD86	CD28	Stimulatory	LGALS9	HAVCR2	Inhibitory
HHLA2	TIMGD2	Stimulatory	LILRB4	UNKNOWN	Inhibitory
ICOSLG	ICOS	Stimulatory	MHC-I	LILRB1	Inhibitory
PVR	CD226	Stimulatory	MUC16	SIGLEC9	Inhibitory
SEMA4A	LILRB2	Stimulatory	MUC16	SIGLEC7	Inhibitory
SEMA4A	TIMD2	Stimulatory	NECTIN2	TIGIT	Inhibitory
SLAMF1	SLAMF1	Stimulatory	NECTIN2	PVRIG	Inhibitory
TIMD4	HAVCR1	stimulatory	NECTIN3	TIGIT	Inhibitory
TNFSF14	TNFRSF14	Stimulatory	PDCD1LG2	PDCD1	Inhibitory
TNFSF18	TNFRSF18	Stimulatory	PVR	TIGIT	Inhibitory
TNFSF4	TNFRSF4	Stimulatory	VSIR	SELPLG	Inhibitory
TNFSF9	TNFRSF9	Stimulatory	SEMA4A	HAVCR2	Inhibitory
NCR3	NCR3LG1	Stimulatory	ST8SIA1	SIGLEC7	Inhibitory
CD48	CD244	Stimulatory	ST8SIA1	SIGLEC9	Inhibitory
BTNL2	UNKNOWN	Inhibitory	TNFRSF14	CD160	Inhibitory
CD24A	SIGLEC10	Inhibitory	TNFSF15	TNFRSF25	Inhibitory
CD274	PDCD1	Inhibitory	TNFSF8	TNFRSF8	Inhibitory
SIRPA	CD47	Inhibitory	NECTIN1	CD96	Inhibitory
CD80	CTLA4	Inhibitory	CD47	SIRPG	Inhibitory
CD86	CTLA4	Inhibitory	UNKNOWN	VTCN1	Inhibitory
CDH1	KLRG1	Inhibitory	UNKNOWN	SIGLEC15	Inhibitory
CDH2	KLRG1	Inhibitory	UNKNOWN	KLRC1	Inhibitory
CEACAM1	HAVCR2	Inhibitory	UNKNOWN	CD276	Inhibitory
FGL1	LAG3	Inhibitory	UNKNOWN	LAIR1	Inhibitory
HHLA2	KIR3DL3	Inhibitory	TNFRSF14	BTLA	Dual functions

**Table 2.** The regulation of immune checkpoint molecules in atherosclerotic cardiovascular diseases.

ICP Molecules	Atherosclerosis Mouse Model	Main Finding/Phenotype	PMID
CTL-4	CTLA-4 transgenic-ApoE^−/−^ mice fed a chow diet	Decreased numbers of effector CD4+ T cells Decreased expression of costimulatory molecules CD80, CD86, and CD28 on CD11c+ dendritic cells Decreased atherosclerotic lesion and intraplaque accumulation of macrophage and CD4+ T cells in the aortic root	27055906
PDL-1	PD-LP1^−/−^/LDLR^−/−^ mice on high-cholesterol diet (HCD) for 10 weeks	Increased CD4+, CD8+ T-cells, and macrophages Increased cytotoxic activity of CD8+ T-cells Increased serum TNF-α levels Increased atherosclerotic lesions	21393583 24691202
LAG3	Bone marrow transplantation (BMT) from wild-type (WT) and LAG3^−/−^ to LDLR^−/−^ mice	Increased levels of TH1 cells and effector/memory T cells, balanced by increased levels of Tregs Increased density of T cells in plaques Plaque size did not change	36636446
GAL-3	6-, 12-, and 20-weeks of HCD fed GAL-3^−/−^/ApoE^−/−^ mice	Decreased atherosclerotic lesion formation at 12 and 20 weeks	23426722
TIGIT	Western diet (WD) fed LDLR^−/−^ mice treated with TIGIT agonist (100 μg) at day 0, 2, 4, 10, 17 and 24 after WD	Suppressed spleen T cell activation and proliferation Elevated DCs percentages and increased activation status Decreased IL-10 production in the blood and spleen Did not affect atherosclerotic lesion development as well as macrophage and collagen content	24376654
TIM-3	WD fed LDLR^−/−^ mice treated with anti-TIM-3 blocking antibody (250 μg) for 3 and 8 weeks	Increased percentages of circulating monocytes and lesional macrophages Increased CD4+ T cells, enhanced their activation status, and reduced percentages of Tregs and Bregs Increased fatty streak formation and atherosclerotic plaque	23990206
GAL-9	12 weeks of HFD fed ApoE^−/−^/GAL-9^−/−^ mice	Decreased aortic atherosclerotic lesion	36459823
CD47	16 weeks WD fed myeloid cell-specific CD47^−/−^ mice	Increased M1 polarization Inhibited efferocytosis and impaired cholesterol efflux Increased atherosclerosis development	34940829
SIRPA	16 weeks WD fed SIRPA^−/−^ and myeloid cell-specific SIRPA^−/−^ mice treated	Induced M2 macrophage phenotype and inhibited necrotic core formation in the arterial wall Stimulates efferocytosis Protected from atherosclerosis in SIRPα^−/−^ in macrophages Reduced cholesterol accumulation	34940829
CD40	Normal chow diet fed CD40 l^fl/fl^/CD4 Cre/ApoE^−/−^	Decreased CD4 T cells Decrease IL-16, IFN-γ but maintain TGF-β and IL-10 Reduced plaque and systemic Tregs Thicker fibrous caps and increased SMC content Decreased atherosclerotic plaque	34145241
CD70	BMT of CD70^−/−^ macrophages to ApoE^−/−^ mice	Impaired the inflammatory capacity of bone marrow-derived macrophages Increased both M1-like and M2-like macrophage markers, and rendered macrophage metabolically inactive and prone to apoptosis CD70-deficient macrophages expressed diminished levels of scavenger receptors and ABC-transporters, impairing uptake of oxidized low-density lipoprotein (oxLDL) and cholesterol efflux Displayed a profound increase in necrotic core size, plaque area, and number of lesional macrophages	27786334
CD27	BMT of CD27^−/−^/ApoE^−/−^ bone marrow to ApoE^−/−^ mice fed with WD for 7 weeks	Reduced the abundance of Treg in blood, lymphoid organs, and the aorta Increased expression of IL-1β and IL-6 in the aorta Increased plaque size and lesional inflammation	29045618
GITR	12 weeks WD fed GITR^−/−^/ApoE^−/−^ mice	GITR^−/−^ApoE^−/−^ monocytes displayed decreased integrin levels, reduced recruitment to endothelium, and produced less reactive oxygen species, less cytokines and had a reduced migratory capacity Decreased necrotic core sizes and thicker fibrous caps Decreased atherosclerotic lesions with reduced CD68+ macrophage content	32728688
OX40L	10 weeks WD fed LDLR^−/−^ mice treated with anti-OX40L blocking antibody for 10 weeks	Reduced Th2 responses, and reduced mast cell presence and activation Increased IL-5 production by T and B1 cells Enhanced atheroprotective oxLDL-specific IgM production Increased IL-33 production by APCs upon OX40L blockade Decreased atherosclerotic lesions	24068673
